# Agronomical and Physiological Behavior of Spanish Hazelnut Selection “Negret-N9” Grafted on Non-suckering Rootstocks

**DOI:** 10.3389/fpls.2021.813902

**Published:** 2022-02-01

**Authors:** Mercè Rovira, J. Francisco Hermoso, Josep Rufat, Valerio Cristofori, Cristian Silvestri, Agustí Romero

**Affiliations:** ^1^Institut de Recerca i Tecnologia Agroalimentàries, Mas Bové, Constantí, Spain; ^2^Institut de Recerca i Tecnologia Agroalimentàries, Parc Científic i Tecnològic Agroalimentari de Lleida, Lleida, Spain; ^3^Dipartimento di Scienze Agrarie e Forestali, Università degli Studi della Tuscia, Viterbo, Italy

**Keywords:** *Corylus avellana* L., clonal rootstocks, physiological traits, yield efficiency, fatty acids profile, oil stability

## Abstract

“Negret” is the most widely planted hazelnut cultivar in Northeastern Spain, where it is highly appreciated by the local kernel marked for its favorable nut traits. Its main disadvantages are the high suckers emission, causing large maintenance costs every year, and its medium-to-low vigor and susceptibility to iron chlorosis. In 2000, a trial to select new vigorous and non-suckering rootstocks for hazelnut was established at IRTA Mas Bové (Spain). The “Negret N-9” selection was grafted onto four clonal rootstocks (“Dundee” and “Newberg” two selections of open-pollinated *Corylus colurna* seedlings, the low suckering cultivar “Tonda Bianca” and the local selection “IRTA MB-69”) and compared to the self-rooted “Negret N-9” as a control. The trial was designed as a randomized complete block with 10 replications and one tree per plot (10 trees per treatment). Plant vigor, suckers emission, yield, and nut and kernel traits have been evaluated over 10 years (2003–2012). During the 2006 to 2010 growing seasons, the qualitative traits of kernels, such as kernel skin color, oil content, and fatty acid profiles, were added to the characterization. Physiological data, such as steam water potential, stomatal conductance, and leaf chlorophyll content, were also evaluated during the 2015 growing season. The results showed that clonal rootstocks had a strong influence on vigor and yield of “Negret N-9.” The “Dundee,” “Newberg,” and “IRTA MB-69” rootstocks showed the highest vegetative growth and the lower suckers emission. The yield was highest in trees grafted on “Dundee” rootstock. In terms of the qualitative traits of kernel which are important to the hazelnut industry, rootstocks increased the oil stability and induced a brown light color in the kernel pellicle versus the brown dark color observed in nuts collected from self-rooted “Negret N-9.” The fatty acids profile was also influenced by the grafting combination. Finally, physiological traits indicated a higher overall performances for “Dundee” rootstock, which was generally found to be the best rootstock for “Negret N-9” in the experimental environment.

## Introduction

Spain is one of the most important hazelnut-producing countries in Europe; during the 2019 growing seasons, its total in-shell production was approximately 12.300 t (FAOSTAT, 2021).

The most widely grown hazelnut cultivar in Spain is “Negret”, and it currently represents approximately 70% of Spanish commercial hazelnut orchards. This cultivar tends to have low vigor and late bearing, is quite susceptible to iron chlorosis, and shows high suckers emission (Tous et al., 1997). These disadvantages may be mitigated by using vigorous rootstocks, which are adapted to calcareous soils, have lower suckering, and can be combined with the use of healthy scions of the main hazelnut cultivars, such as the virus-free clonal selection “Negret N-9” (Rovira et al., 2006), released by the IRTA Mas Bové, Constantí, Tarragona (Spain), and which resulted to be more yielding than the standard “Negret” (Aramburu and Rovira, 1998).

The natural habitat of the European hazelnut (*Corylus avellana* L.) is a large multi-stemmed shrub, which annually produces suckers from buds located at the base of the trunk or tree stump. The suckers are the natural replacement for old, dead, or diseased stems. To facilitate the management in commercial orchards, growers in several producing countries [the United States (in Oregon), France, Chile, and to a lesser extent Spain and Italy] train hazelnut trees to a single trunk. This training system makes it necessary to eliminate the suckers that continuously appear each growing season. This sucker removal becomes a major cropping operation, requiring four to five herbicide sprays per year and occasionally hand sucker removal during the winter, representing a significant increase in time and money spent (Lagerstedt, 1975; Tous et al., 1994; Radicatti et al., 1997; Cerović et al., 2009; Fideghelli and De Salvador, 2009; Silvestri et al., 2021a). This current state of the art may be improved by using non-suckering rootstocks in commercial hazelnut orchards, as has been reported by different authors (Molnar, 2011; Ellena et al., 2014; Cristofori et al., 2017; Silvestri et al., 2021b). Although interest has been widely expressed by the hazelnut community in the use of non-suckering rootstocks, field evaluations on this topic are still limited, and the few trials that exist are still in progress.

The breeding activities aimed to release hazelnut rootstocks, in addition to the patenting of new cultivars, started in 1968 at Oregon State University (United States). In nursery rows, *Corylus colurna* L. seedlings were obtained by open pollination of *C. avellana*, and growth habits similar to those of the European hazelnut were selected and propagated by layering. Over 20 years, about 150 putative inter-specific hybrids were selected from among 20,000 seedlings raised, and two of these selections, named “Newberg” (USOR 7-71) and “Dundee” (USOR 15-71), were released as clonal rootstocks (Lagerstedt, 1993a). Both selected non-suckering hybrids provide high vigor to the scion of European hazelnut cultivars when grafted onto them. Despite their promise, in the past these clonal rootstocks have been used infrequently in the United States as well as in other hazelnut-producing countries. Hazelnut growers in Oregon (United States) did not use this innovation in grafted plant material, such as “Dundee” and “Newberg” were highly susceptible to the local disease Eastern Filbert Blight (EFB) caused by the ascomycete *Anisogramma anomala* (Pinkerton et al., 1992), as this fungal disease is the main drawback for hazelnut growing in the United States (Mehlenbacher, 1991). Nevertheless, hazelnut growers in Northeast Spain have recently highlighted their interest in these rootstocks, and about 200 ha of commercial orchards have been planted using trees grafted on “Dundee” rootstock that now is routinely propagated by some Spanish nurseries (Rovira, personal communication).

Moreover, also in Italy some research centers have started to establish trials for testing different Italian cultivars grafted on “Dundee” rootstocks and new selected seedlings obtained by crossing *C. colurna* L. × *C. avellana* L. (Valentini et al., 2007, 2009), and in Craiova University (Valcea, Romania), where in 2016, three local varieties named “Cozia,” “Uriase de Valcea,” “Valcea,” and some standard cultivars as “Hall’s Giant,” “Romavel,” and “Tonda Gentile delle Langhe” were grafted onto “Dundee” and “Newberg” rootstocks and their agronomic behavior is under study (Botu, personal communication).

*Corylus colurna* L. and perhaps hybrids of *C. colurna* × *C. avellana* usually show high drought tolerance due to their deeper roots than *C. avellana*, and their non-suckering aptitudes give a high potential to this plant material as a rootstock (Botta et al., 2019). Nevertheless, *C. colurna* seedlings grafted with scions of the main European hazelnut cultivars show some disadvantages, such as the seed germination, which in nurseries usually takes 2 years to germinate, where the seedlings often require 2 additional years to properly grow before reaching a suitable size for grafting. According to these issues, their use in commercial nurseries is still limited. Despite the disadvantages observed for seedlings of *C. colurna*, in nurseries, to be used as rootstock, this plant material is widely used in Serbia (Korac et al., 1997; Cerović et al., 2009; Nikolova, 2009; Ninić-Todorović et al., 2009, 2012) since it does not produce suckers, gives long life to the grafted trees, shows high resistance to frost and drought, and it seems adaptable to a wide range of soils (Miletic et al., 2009).

In the past some vigorous and low suckering cultivars of *C. avellana*, have been also tested as rootstocks in the United States (Lagerstedt, 1975), in Italy (Baratta et al., 1990), and in Spain (Tous et al., 1997). The trial carried out in Spain released the clonal rootstock “MB-69” selected by seedlings of “Tonda Bianca” (Tous et al., 1997). In Karaj (Iran), local genotypes of *C. avellana* tolerant to drought and low humidity have been selected as rootstocks (Salimi and Hoseinova, 2012). Furthermore, in the Chilean Research Center “Instituto de Investigaciones Agropecuarias – INIA” located in Temuco (Araucanía region, southern Chile), the clone BA-5 selected by “Chilean Barcelona” cultivar has been tested as a promising non-suckering rootstock for European hazelnut (Ellena et al., 2014).

The European hazelnut (*C. avellana* L.) is considered a species sensitive to water stress and usually shows a low capacity of stomatal regulation (Girona et al., 1994; Cristofori et al., 2014; Gispert et al., 2015). This physiological inefficiency can negatively influence the foliar photosynthetic activity and promotes a poor nut filling when drought condition becomes extended (Tombesi, 1994; Dias et al., 2005). Seedlings of *C. colurna* and hybrids of *C. colurna* × *C. avellana* could optimize this physiological trait in grafted trees for commercial purposes thanks to their root systems, which are deeper than those of self-rooted cultivars of *C. avellana*.

On this framework, our research aimed to study for a long period the agronomic and physiological behavior of the Spanish hazelnut cultivar “Negret N-9” grafted on four putative clonal rootstocks in comparison to self-rooted trees of the same cultivar.

## Materials and Methods

### Plant Material

The trial was carried out over the period 2000–2015 in the experimental orchard located at IRTA – Mas Bové Research Centre (Constantí, Tarragona, Northeastern Spain) (41°10′9′′N, 1°10′28′′E, altitude 110 m a.s.l.) in a loamy–sandy soil texture (United States Department of Agriculture) classified as Typic Xeropsamments (Comisión del Banco de Datos de Suelos y Aguas, 1983). It is an alkaline soil (active limestone 4%), with low organic matter content (1% at 0–50 cm in soil depth) and medium-to-high active lime content (<7%), without an excess of salinity and low phosphorus and potassium content. The area is characterized by a Mediterranean coastal climate, and data of maximum and minimum air temperatures and rainfall were collected by a thermometric station located near the experimental orchards (Supplementary Figure 1). Average annual rainfall and reference evapotranspiration (Et_0_) over the trial period were 580 and 1041 mm, respectively.

The trial was established by planting virus-free trees of the clone “Negret N-9” selected by IRTA, since it is characterized by low vigor, susceptibility to iron chlorosis, and high suckers emission. Trees of this clone were grafted onto four different clonal rootstocks: “Dundee” and “Newberg,” two selections from Oregon State University (United States) (Lagerstedt, 1993b), the Italian cultivar “Tonda Bianca” selected in the past in Campania region (Eynard et al., 1972), and the IRTA’s selection “MB-69” (Tous et al., 1997). These grafting combinations were compared with self-rooted trees of the clone and inserted in the trial as controls. Grafting material was collected from mature trees, and whip grafted trees were produced using a “hot callusing pipe” system in a greenhouse (Lagerstedt, 1981). The “Negret N-9” self-rooted trees were obtained by layering from donor trees as described in the literature (Germain and Sarraquigne, 2004).

The experiment was designed as a randomized complete block with 10 replications and one tree per plot (10 trees per treatment). The trees were spaced at 6.0 m × 3.5 m and trained into a vase-shaped single-trunk form. The experimental orchard was managed with a natural green cover crop according to the rules of the local hazelnut Integrated Production System. Furthermore, due to the low annual rainfall, a drip irrigation supply was ensured during the summer period, and it consisted of a pipe of 20 mm and self-compensated drippers with 4 L of water per hour and a spacing of 70 cm in the drip line. The volumes of water supply were calculated as ETc = ETo × Kc, where ETo is the class A evaporation and Kc is the crop coefficient for hazelnut in Tarragona (Spain), as reported in the literature (Girona et al., 1994).

### Agronomic Traits

The agronomical data were recorded annually over a 10-year period (2003–2012) on all the grafted and self-rooted trees by measuring tree height (centimeters), trunk cross-sectional area [TCSA (square centimeters)] at 20 cm above the ground, total number of suckers per tree, canopy volume (cubic meters), and yield (kilograms) expressed as total in-shell nuts per tree. In 2012, when the trees reached the maximum available space between them, the yield efficiency (YE) (the ratio between cumulative yield and TCSA) was calculated.

### Nut and Kernel Traits

Several traits including nut weight (grams), kernel weight (grams), percent kernel [(kernel weight/nut weight) × 100], kernel size >12 mm (percent), and the main nut defects, such as blanks, shriveling, a glassy appearance, and inner kernel cavity (hulls inside the kernel when it is opened), were investigated over the years by using randomized samples of 50 nuts per tree. Skin color components, lightness (L*), reddish (a*), and yellowish (b*), were measured using a spectrophotometer (MINOLTA Model M3500) on the samples collected in 2009 and 2010. In addition, kernel samples collected in the the 2010 growing season were taken and placed in an oven at 175 ± 1°C for 30 min, and they were then cooled at room temperature before being peeled by hand. After pellicle removal, the kernels were classified according to roasting index RI1 (incidence of roasted kernels peeled in more than 95%) and roasting index RI2 (incidence of roasted kernels peeled in more than 50%).

Fatty acids profiles and oil stability were investigated for 5 consecutive years (2006–2010). Briefly, the fatty acid methyl esters (FAMEs) were prepared by trans-esterification with KOH 0.5 M, following the official method UNE-EN ISO 5509:2000. The FAMEs (1 mL) were separated using a gas-chromatograph (HP 6890; Agilent Technologies, Barcelona, Spain) equipped with an FID detector and a capillary column 30 m lenght and 0.25 mm diameter (HP-Innowax, Agilent Technologies). The carrier gas was helium, and the flow rate was 1 mL/min. The injector and detector temperatures were +220 and +275°C, respectively. The FAME identification was based on retention time relative to those of a standard FAME mixture (Sigma-Aldrich, Madrid, Spain). Oil stability was measured by Rancimat method, using a Rancimat 617 series 09 Metrohm, working at +120 ± 1°C and 20L/h of air flow; the stability values were expressed in hours.

### Physiological Measurements

The stem water potential, chlorophyll index (SPAD), and stomatal conductance were measured during the 2015 growing season. Stem water potential was monitored with a pressure chamber (Scholander et al., 1965) following the recommendations of Turner and Long (1980). Briefly, readings were taken with a plant status console (Model 3005, Soil Moisture Equipment Corp., Santa Barbara, CA, United States). Stem water potential was measured two times (June 22 and August 28), on two selected leaves of both north and south orientation, covered with a bag (shaded leaves) before measuring the pressure. The chlorophyll Index was measured threefold (May 21, June 22, and August 28) in 12 leaves per tree (three leaves for each orientation), by non-destructive instruments (SPAD-502, KONICA MINOLTA). Stomatal conductance was measured twice (June 22 and August 28) on two leaves per tree, with a steady-state porometer (model LI-1600, Li-Cor, Lincoln, NE, United States). All these physiological data were collected at noon. In addition, leaf drop in late summer–autumn (four labeled branches per tree) were counted once a week, from the first of September to the middle of December 2015. The phenological phases at physiological measurement times were: just before fecundation (May 21), the kernel fills 30% of the shell (June 22), and near harvest time (August 28). All physiological measurements were taken in three trees per treatment of the trial. For stem water potential and stomatal conductance, 6 leaves per treatment (1 tree/repetition and 2 leaves per tree) were run and a total of 30 leaves per day were used to avoid a time measurement beyond 1 h.

### Statistical Analysis

The effect of different rootstocks compared to the self-rooted control were evaluated and data have been subjected to one-way ANOVA; mean separation was done using Duncan’s multiple range test (*P* ≤ 0.01). Principal component analysis (PCA) was performed using all the quantitative variables to assess their relationships. Data processing was performed with SAS (version 9.4; SAS Institute, Cary, NC, United States).

## Results

### Climatic Data Over the Period 2003–2012

Data of maximum and minimum air temperature and rainfall, collected by the thermometric station over the period 2003–2012 are presented in Supplementary Figure 1. Notably, the mean rainfall detected over the period was approximately 560 mm per year, showing a limited number of rainfall events in June and July, which was identified as the timeframe during the growing seasons in which to focus on the irrigation supply.

### Agronomic Traits

All agronomic traits investigated showed significant differences related to the type of grafting combination and year of observation. Suckers emission differed significantly depending on the rootstock; as a comparison, what was observed in the self-rooted “Negret N-9.” Rootstock with the lowest suckers emission was the selection “MB-69” with an average of 2.77 suckers per tree, and it was followed by the rootstocks “Newberg” and “Dundee,” which showed values of 3.12 and 4.07, respectively (Table 1). The highest sucker’s emission rootstock was the self-rooted “Negret N-9” that yearly produced an average of 25.44 suckers per tree.

**TABLE 1:** Effects of rootstock on suckers production, trunk cross-sectional area (TCSA), tree height, tree diameter, and canopy volume, in “Negret N-9” hazelnut cultivar planted in 2000, at 6 × 3.5-m (19.7 × 11.48 ft) tree spacing at the Institute of Agriculture and Food Research and Technology (IRTA)-Mas Bové Station (Constantí, Spain).

Rootstock	Suckers 2003–2011 (no/year)^z^	TCSA 2012 (cm^2^)^y^	Tree height 2012 (cm)^x^	Tree diameter 2012 (cm)	Canopy volume 2012 (m^3^)^x^
“Dundee”	4.07 c^w^	234.91 a	317.0 a	408.95 a	28.42 a
“Newberg”	3.12 c	209.07 a	305.25 ab	389.81 ab	24.56 ab
“Tonda Bianca”	9.18 b	202.47 a	297.78 ab	353.67 cd	19.56 c
“MB-69”	2.77 c	202.98 a	292.0 ab	371.40 bc	21.09 bc
Self-rooted	25.44 a	105.58 b	271.11 b	343.17 d	16.75 c

*^z^Mean number of suckers produced per year.*

*^y^TCSA measured above the graft union. For own-rooted trees at 20 cm (7.9 inches) above the ground level; I cm^2^ = 0.1550 inch^2^.*

*^x^1 cm = 0.3937 inch, 1 m^3^ = 35.314 ft^3^.*

*^w^Means within a column followed by the same letter are not significantly different by Duncan’s multiple range test at P ≤ 0.05.*

All grafted trees were more vigorous than self-rooted ones (Table 1), and the values of TCSA (cm^2^) tree height (cm), tree diameter (cm), and canopy volume (m^3^) recorded in 2012 were significantly higher especially for “Negret N-9” grafted on “Dundee.”

Results presented in Table 2 show the cumulative yield expressed as kilograms of in-shell nuts per tree, recorded during the first cropping years when the trees were still young (2003–2006), the cumulative yield recorded during the whole period of the trial (2003–2012), and YE. In the first cropping years, trees of “Negret N-9” grafted on “Dundee” and “MB-69” rootstocks showed the highest cumulative yield (10.23 and 9.63 kg per tree, respectively), as also confirmed by statistical analysis. Significant differences were also observed for the total cumulative yield (2003–2012), where trees grafted on “Dundee” rootstock had the highest average value (41.91 kg per tree) and followed by “Newberg” (34.91 kg per tree), whereas the own-rooted “Negret N-9” showed the lowest value (26.91 kg per tree). YE calculated as the cumulative yield from 2003 to 2012 and related to the TCSA detected at the end of growing season 2012, was highest for the lowest vigorous tree of self-rooted “Negret N-9.”

**TABLE 2:** Effects of rootstock on early bearing, cumulative yield, and yield efficiency, in “Negret N-9” hazelnut cultivar planted in 2000, at 6 × 3.5-m (19.7 × 11.48 ft) tree spacing at the Institute of Agriculture and Food Research and Technology (IRTA)-Mas Bové (Constantí, Spain).

Rootstock	Cumulative early bearing 2003–2006 (kg/tree)^z^	Cumulative total yield 2003–2012 (kg/tree)^y^	Cummulative yield efficiency (Kg/cm^2^)
“Dundee”	10.23 a^w^	41.91 a	0.1829 b
“Newberg”	7.73 b	34.91 b	0.1714 bc
“Tonda Bianca”	6.99 b	25.64 d	0.1357 c
“MB-69”	9.63 a	31.55 bc	0.1593 bc
Self-rooted	7.44 b	26.91 cd	0.2626 a

*^z^Total crop (in-shell nuts) per tree from 2003 to 2006; 1 kg = 2.2046 lb.*

*^y^Total crop (in-shell nuts) per tree from 2003 to 2012.*

*^x^Total crop (in-shell nuts) per tree from 2003 to 2012 related to trunk cross-sectional area (TCSA), measured above the graft union, for own-rooted trees measured at 20 cm (7.9 inches) above the ground level, at the end of the year 2012; 1 kg cm^–2^ = 14.2233 lb/inch^2^.*

*^w^Means within a column followed by the same letter are not significantly different by Duncan’s multiple range test at P ≤ 0.05.*

**FIGURE 1:**
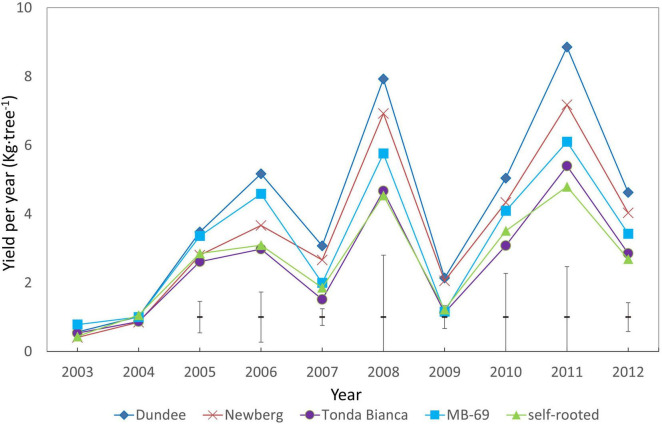
Yield (in-shell nuts) of different trees materials represented by “Negret N-9” grafted on “Dundee,” “Newberg,” “MB-69,” and “Tonda Bianca” rootstocks, respectively, and compared to self-rooted “Negret N-9.” Data recorded from 2003 to 2012. Error mean square for each year, are included.

A thorough analysis of the yearly yield of the trial showed that, since 2005, large differences were observed for the different grafting combinations; these were maintained until the last year of the trial (2012). As showed in Figure 1, trees grafted on “Dundee” and similarly to those of “Newberg,” stand out for their nut production when compared to the other rootstocks and to the self-rooted “Negret N-9,” which was also confirmed to be the lowest producing thesis.

### Nut and Kernel Traits

The nut and kernel traits, investigated throughout the trial from the first year of tree production, are grouped in Table 3. The nut samples collected from the self-rooted “Negret N-9” trees were slightly higher in weight and size when compared to those collected from the grafted “Negret N-9” trees, whereas no differences were observed for the trait percent kernel (kernel/nut ratio) that on average was about 49% for all theses.

**TABLE 3:** Effects of rootstock on nut and kernel weight, kernel percentage, kernel size [>12 mm (0.47 inch)], and inner kernel cavity, in “Negret N-9” hazelnut cultivar planted in 2000, at 6 × 3.5-m (19.7 × 11.48 ft) tree spacing at the Institute of Agriculture and Food Research and Technology (IRTA)-Mas Bové (Constantí, Spain).

Rootstock	Nut wt (g)^z^	Kernel wt (g)	Kernel percentage (%)^y^	Kernels >12 mm (%)	Inner kernel cavity (%)
“Dundee”	1.81 b^x^	0.89 b^z^	49.17 a	20.91 b	37.85 a
“Newberg”	1.79 b	0.89 b	49.72 a	20.12 b	34.94 ab
“Tonda Bianca”	1.79 b	0.88 b	49.16 a	19.38 b	33.62 bc
“MB-69”	1.81 b	0.89 b	49.17 a	12.21 b	33.82 bc
Self-rooted	1.84 a	0.91 a	49.45 a	30.49 a	30.33 c

*Mean values from 2003 to 2012.*

*^z^1 g = 0.0353 oz.*

*^y^(kernel weight/nut weight) × 100.*

*^x^Means within a column followed by the same letter are not significantly different by Duncan’s multiple range test at P ≤ 0.05.*

Referring to nut and kernel defects, no significant differences were observed in blanks, shrivel, or kernels with glassy appearance (data not shown), highlighting that the rootstock does not influence the incidence of these nut and kernel disorders. Nevertheless, nuts collected from self-rooted “Negret N-9” trees were affected by lower inner kernel cavity than those collected from grafted trees (Table 3). Significant differences in skin kernel (pellicle) color were also observed, and, as shown in Table 4, higher values of lightness (*L* = 29.46) were found in kernels of the grafted trees onto “Dundee” rootstock, whereas lower entity of lightness was recorded for kernels of the self-rooted trees (*L* = 27.50). These differences in lightness of the kernel pellicle are mainly influenced by the yellow component of the pellicle color (*b*) that was significantly higher in kernels of the trees grafted on “Newberg” (*b* = 14.68), “Dundee” (*b* = 14.41) and “Tonda Bianca” (*b* = 14.48), respectively, whereas the kernels harvested from self-rooted trees showed the lowest mean values of this trait (*b* = 13.52). No difference emerged for the parameters roasting index RI1 and RI2 (data not shown) confirming that rootstock does not affect this technological parameter of the nuts, which is demonstrated to be highly genotype-dependent in a manner similar to the nut and kernel defects (Mehlenbacher et al., 1993).

**TABLE 4:** Effects of rootstock on skin kernel color in “Negret N-9” hazelnut cultivar planted in 2000, at 6 × 3.5-m (19.7 × 11.48 ft) tree spacing at the Institute of Agriculture and Food Research and Technology (IRTA)-Mas Bové (Constantí, Spain).

Rootstock	L: lightness	b: yellow component	a: red component
“Dundee”	29.46 a^z^	14.41 ab	8.51 a
“Newberg”	28.27 ab	14.68 a	8.56 a
“Tonda Bianca”	28.65 ab	14.48 ab	8.47 a
“MB-69”	28.23 bc	13.77 bc	8.32 a
Self-rooted	27.50 c	13.52 c	8.31 a

*Mean values from 2009 to 2010.*

*^z^Means within a column followed by the same letter are not significantly different by Duncan’s multiple range test at P ≤ 0.05.*

Qualitative traits of the hazelnut kernels are highly influenced by their oil content that usually reaches 60–65% of the seed dry weight and consist of approximately 80% oleic acid (Cristofori et al., 2015). Notable differences were recorded for oil stability among the grafting combinations. Oil extracted from kernels of “Negret N-9” grafted on “Dundee,” “Newberg,” and “Tonda Bianca” showed a higher oil stability (6.80, 6.45, and 6.92 h, respectively) when compared to that of nuts collected from self-rooted trees, which showed mean values of 5.67 h (Table 5). The fatty acids composition was analyzed from the extracted oil by the kernels harvested from the representative trees of the grafting combinations under test conditions, and the incidence of the main fatty acids (palmitic, oleic, and linoleic acids) is also shown in Table 5. Although slight differences were observed for these qualitative traits such as for palmitic acid expressed as mean value of 5 cropping years (from 2006 to 2010), which was lower than 6% in self-rooted thesis, it was not possible to establish any relationship between fatty acids composition and plant vigor, yield, or technological kernel traits.

**TABLE 5:** Effects of rootstock on kernel oil stability (hours at 120°C) and relative fatty acid composition (%) of “Negret N-9” hazelnut cultivar planted.

		Major fatty acid composition (%)^z^		
Rootstock	Oil stability (h)	C16:0	C18:1	C18:2
“Dundee”	6.80 a^y^	6.1 ab	76.8 ab	14.73 b
“Newberg”	6.45 a	6.2 ab	75.2 b	16.48 a
“Tonda Bianca”	6.92 a	6.31 a	77.9 a	14.45 b
“MB-69”	6.33 ab	6.0 b	76.4 ab	15.41 ab
Self-rooted	5.67 b	5.9 b	75.9 ab	15.76 ab

*Mean values from 2006 to 2010.*

*^z^C16:0 = palmitic acid; C18:1 = oleic acid; C18:2 = linoleic acid.*

*^y^Means within a column followed by the same letter are not significantly different by Duncan’s multiple range test at P ≤ 0.05.*

### Physiological Traits

**FIGURE 2:**
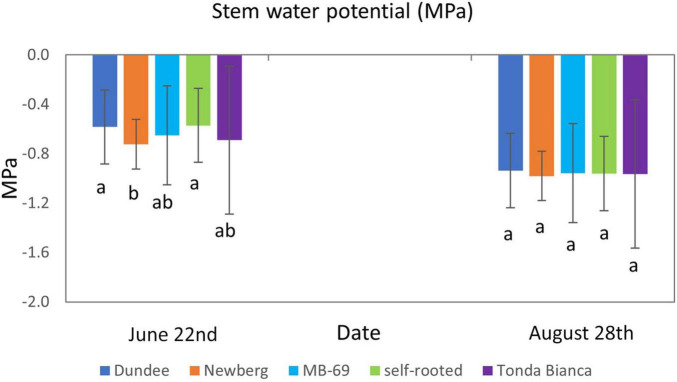
Stem water potential (Ψst) (MPa) of “Negret N-9” trees grafted on “Dundee,” “Newberg,” “MB-69,” “Tonda Bianca” rootstocks, and self-rooted (control). Data were evaluated in 2015, on June 22 and August 28. Means within a date followed by the same letter are not significantly different by Duncan’s multiple range test at *P* ≤ 0.05.

Seasonal evolution of the stem water potential (Ψst) monitored in two different eco-physiological stages of the trees during the 2015 growing season (late June and late August) clearly showed higher Ψst for trees grafted onto “Dundee” that were characterized by values of −0.58 and −0.93 MPa in late June and late August, respectively, as well as observed in self-rooted trees that showed values of −0.57 and −0.96 MPa, in the same times of investigation. Conversely, trees grafted on “Newberg” showed the lowest values of Ψst (−0.72 and −0.98 MPa, respectively), while the trees grafted onto “MB-69” and “Tonda Bianca” were characterized by intermediate Ψst values (−0.65 and −0.95 MPa for “MB-69” and −0.69 and −0.96 MPa for “Tonda Bianca,” respectively), especially for measurements carried out in late June when the trees were distinguished by the phase of fruit development and early kernel filling, which significantly influences their eco-physiological behavior (Cristofori et al., 2015). Moreover, while the differences observed during the first date of measurements were affected by the rootstock, no significant differences were emerged for this trait at near harvest time (August 28), as shown in Figure 2.

**FIGURE 3:**
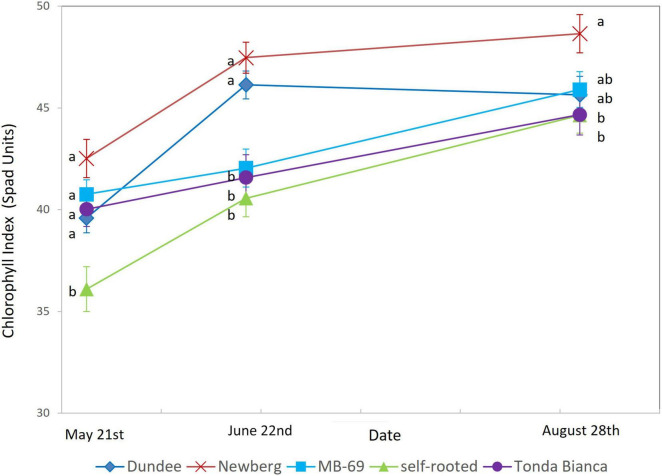
Chlorophyll index of “Negret N-9” trees grafted on “Dundee,” “Newberg,” “MB-69,” “Tonda Bianca” rootstocks, and self-rooted (control). Data were evaluated in 2015, on May 21, June 22, and August 28. Means within a date followed by the same letter are not significantly different by Duncan’s multiple range test at *P* ≤ 0.05. Error mean squares are included.

Seasonal patterns on chlorophyll index (Figure 3) showed lower values for trees grafted onto “Tonda Bianca” and for self-rooted “Negret N-9” when compared to those recorded for the other grafting combinations, with particular reference to trees grafted onto “Newberg”, which showed the highest values throughout the whole measurement campaign carried out in 2015 (43, 47.5, and 48.6 on May 21, June 22, and August 28, respectively).

**FIGURE 4:**
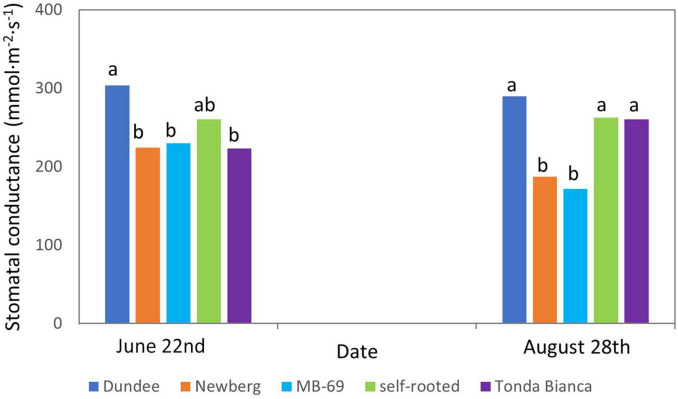
Stomatal conductance of “Negret N-9” trees grafted on “Dundee,” “Newberg,” “MB-69,” “Tonda Bianca” rootstocks, and self-rooted (control). Data were evaluated in 2015, on June 22 and August 28. Means within a date followed by the same letter are not significantly different by Duncan’s multiple range test at *P* ≤ 0.05. Error mean squares are included.

The leaf stomatal conductance (Figure 4), measured at the same time of Ψst measurements, gave mean values of 303.9 mmol/m^2^/s for “Negret N-9” grafted on “Dundee” at the end of spring (June 22), which were significantly higher than those recorded for the other grafting combinations that grouped at mean values of approximately 230 mmol/m^2^/s, whereas the self-rooted trees showed intermediate mean values (260 mmol/m^2^/s). The leaf stomatal conductance showed higher variability when determined at harvest time (August 28) in comparison to those recorded in late June. In detail, the trees grafted onto “Dundee,” “Tonda Bianca,” and the self-rooted controls were characterized by an average stomatal conductance significantly higher than that detected for “Newberg” and “MB-69.” Furthermore, trees grafted on “Dundee” rootstock were distinguished from other grafting combinations by its higher conductance values, which remained high even near the end of the growing season (289 mmol/m^2^/s in “Dundee,” 260 mmol/m^2^/s in “Tonda Bianca” and self-rooted trees, 188 mmol/m^2^/s in “Newberg,” 171 mmol/m^2^/s in “MB-69”).

**FIGURE 5:**
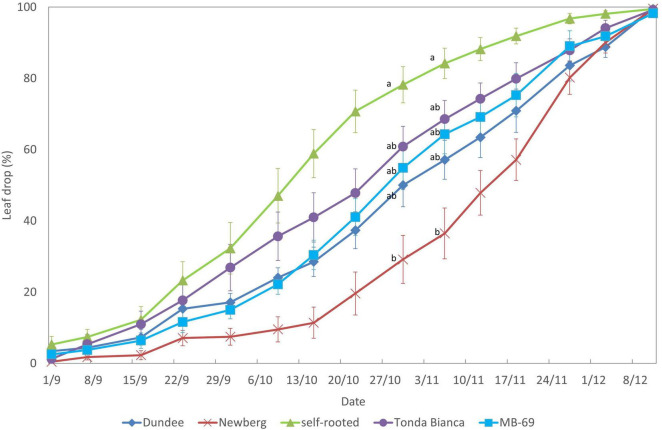
Leaf drops of “Negret N-9” trees grafted on “Dundee,” “Newberg,” “MB-69,” “Tonda Bianca” rootstocks, and self-rooted (control). Data were evaluated in 2015, weekly from September 1 to December 11. Means within a date followed by the same letter are not significantly different by Duncan’s multiple range test at *P* ≤ 0.05. Error mean squares are included.

From the end of September of the same growing season, differences in leaf drop were recorded, and the earliest incidence of leaf fall was observed in self-rooted trees that at the beginning of November showed more than 70% of fallen leaves on the ground (Figure 5). Conversely, the slowest leaf drops were observed for trees grafted on “Newberg” rootstock at the same time (early November), showing a significantly lower incidence of leaf drop, with mean values of 40%. The other grafting combinations were characterized by intermediate leaf drop incidence values during the observation period, as shown in Figure 5.

## Discussion

Results of this trial confirm for all grafting combinations of the trial the distinctive feature of hazelnut that when mature, it is characterized by a large number of nuts in on-crop years, followed by a low number of nuts in off-crop years (Figure 1). This is mainly due to the biennial bearing that affects this species. The years 2007 and 2009 were off-crop years not only for the production in this trial but also for all hazelnut production in Spain (MAPAMA, 2021). This physiological phenomenon, commonly known as “alternate bearing,” is highly genotype-dependent and can be strongly influenced by many factors, including agronomic interventions such as irrigation, nutrition, and pruning (Azarenko et al., 2005), while there is still limited evidence of a possible effect of rootstock on alleviating this physiological issue in hazelnut.

The duration of the trial was over a long period of time to prove that the grafting combinations were significantly different for their agronomical and nut traits in comparison to those detected for self-rooted trees of “Negret N-9.” According to the literature, the low sucker’s emission of “Negret N-9” grafted on “Dundee,” “Newberg,” and “MB-69” is a desired feature for planting large new hazelnut orchards (Lagerstedt, 1975; Tous et al., 1994; Radicatti et al., 1997; Cerović et al., 2009; Fideghelli and De Salvador, 2009; Silvestri et al., 2021b). The use of these low-suckering rootstocks may improve the management and mechanization of the hazelnut orchard and may contribute to saving labor and cost for the seasonal suckers’ removal that is still carried out by hand or spraying herbicides for more time per growing season (Serdar and Akyuz, 2018). Furthermore, avoiding the use of herbicides for suckers control in orchard management will be more environmental friendly.

The high vigor displayed by grafted trees, already observed in the early years of the trial when the young trees were still unproductive (Tous et al., 2009; Rovira et al., 2014), were also confirmed by other trials, as reported in the literature for other grafting combinations (Lagerstedt, 1993a; Tous et al., 1997). In addition, a trial focused on the use of seedlings of *C. colurna* as rootstock, which is routine in Serbia, confirmed that the grafted trees were more vigorous than the same self-rooted local cultivars used for grafting (Cerović et al., 2009; Miletic et al., 2009).

Farinelli et al. (2018) obtained some preliminary results in a trial established in central Italy when grafting some Italian hazelnut cultivars (“Tonda Gentile delle Langhe,” “Tonda di Giffoni,” “Tonda Romana,” and “Tonda Francescana”) on *C. colurna* seedlings; they noticed the vigor of the grafted trees was lower than that of the self-rooted trees. It is probably due to the own “genotypic effect” of each seedling used as rootstock, which is not as uniform as those imparted by the clonal rootstocks used in our trial. This assumption is supported by other evidence, as reported in Oregon (United States) where mature trees in commercial orchards established a practice of grafting local varieties on seedlings of *C. colurna*, which were more unstable in terms of the yearly nut yield when compared to self-rooted trees (Thompson et al., 1992).

Trials carried out in Serbia testing seedlings of *C. colurna* as hazelnut rootstocks highlighted how grafted trees increased their cumulative yield when compared to those self-rooted (Miletic et al., 2009).

In our trial, the higher cumulative yield recorded in “Negret N-9” grafted on “Dundee” rootstock strengthened what was observed during the first study period of the trial (Tous et al., 2009; Rovira et al., 2014).

The rootstock influence on hazelnut eco-physiology was recently confirmed by Ellena et al. (2014); in their trial carried out in Chile, they noticed that the Italian cultivar “Tonda di Giffoni” grafted on the putative rootstock BA5 obtained by clonal selection of cultivar “Chilean Barcelona,” shortened the unproductive stage of the grafted trees, and increased the number of catkins when compared to “Tonda di Giffoni” self-rooted trees. The rootstocks tested in our trial influenced the nut and kernel traits, similarly to what was observed at the early stage of the same planting by Tous et al. (2009) and Rovira et al. (2014), and the same was also noticed in a trial carried out in Northeastern Spain by Tous et al. (1997) that tested some genotypes of *C. avellana* as rootstocks, nut and kernel weight and kernel size was higher in self-rooted trees. These findings are in contrast with those obtained by Miletic et al. (2009) who, in their test carried out grafting the cultivars “Rimski,” “Istarski Dugi,” “Tonda Gentile Romana,” and “Cosford” on seedlings of Turkish hazel (*C. colurna* L.), noticed that the nut and kernel weights were higher in the grafted trees compared with self-rooted trees.

Dark kernel skin color and its astringency, which represents an unpleasant trait in hazelnut consumption, seems to be related to high levels of red and low levels of lightness and yellow components, as reported for almonds (Gou et al., 2000). Based on this correlation and according to our findings (Table 4), the kernels of “Negret N-9” harvested from self-rooted trees showed significantly darker skin color than those from grafted trees on “Dundee,” “Newberg,” and “Tonda Bianca,” suggesting that kernels from grafted trees would have more acceptance for consumers.

Significant differences were observed for both oil stability and fatty acid composition. Kernels from trees grafted on “Tonda Bianca” showed the highest oil stability and oleic acid content, whereas kernels from self-rooted trees presented the lowest levels for these characteristics (Table 5). However, such differences represent less than 15% for oil stability and 5% for oleic acid content.

**FIGURE 6:**
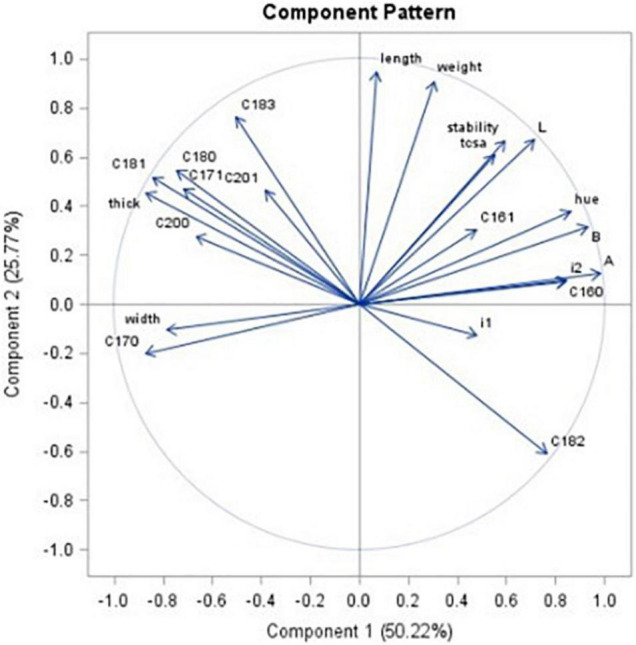
Principal component analysis. Component 1 explains 50.22% of total variability; Component 2 explains 25.77% of variability. The figure shows the loadings for each quantitative variable.

Principal component analysis (Figure 6) aimed to explain 75% of total variability with two principal components. It was pointed out that there is a close relationship among TCSA, oil stability, and lightness (L*), suggesting that the best tree performance shows the highest oil stability.

The low values of stem water potential recorded in the trial, at harvest time, suggested that the trees tended to be affected by water stress. In more detail, “Negret N-9” grafted on “Newberg” rootstock showed the lowest values that reflect on more water stress aptitude (Figure 2). Nevertheless, data obtained from “Negret N-9” grafted on “Dundee” were similar to those recorded for self-rooted trees, and since “Negret N-9” grafted on “Dundee” were more vigorous and productive than self-rooted “Negret,” as shown in Tables 1, 2, it can be deduced that grafted trees on “Dundee” rootstock were less affected by water stress. Apart from these results, further experiments are needed to obtain a clearer pattern of water potential.

In Portugal, values of stem water potential were tested on different irrigation treatments applied to the local cultivar “Grada de Viseu” ranged between −1.4 and −1.6 MPa (Dias et al., 2005), while in a comparative trial between trees trained at single trunk versus multi-stemmed shrubs of the cultivars “Butler” and “Segorbe,” the tested trait ranged between −1.1 and −1.3 MPa without differences among cultivars and training systems (Gonçalves et al., 2009). In addition, in Chile, some studies were conducted comparing the stem water potential of three cvs: “Barcelona,” “Tonda di Giffoni,” and “Tonda Gentile delle Langue” (Grau and Sandoval, 2009), all three cultivars showed a similar trend in the midday water potential, and there were no differences between cultivars. In Italy, some researchers noticed different cultivars tested in the same environment showed different values of stem water potential at the same time of investigation, with the cultivar “Tonda Gentile delle Langhe” that showed mean values of −1.16 MPa, whereas the cv “Tonda di Giffoni” and “Tonda Gentile Romana” had values of −1.01 and −0.84 MPa, respectively (Cincera et al., 2019).

The leaf chlorophyll index is an eco-physiological parameter poorly used in hazelnut, especially for field measurements (Silvestri et al., 2019), whereas it could be effectively related to some leaf monitoring such as early occurrences of iron chlorosis, which frequently affects hazelnut trees. The SPAD measurements carried out on May 21, June 22, and August 28 indicated that leaves of self-rooted “Negret N-9” were less green, as confirmed by lower SPAD values, when compared to those detected for other grafting combinations; this is in contrast to the cultivar “Negret N-9” grafted on “Newberg” rootstock, which showed the highest values (Figure 3). This finding may indicate that grafted trees have a higher leaf chlorophyll content and thus higher photosynthetic potential. In addition, the higher SPAD values recorded for grafted trees on the rootstocks “Newberg,” “Dundee” and “MB-69,” may be related to the higher tolerance to iron chlorosis in comparison to self-rooted trees of “Negret N-9,” such as recently reported by Rovira (2021).

The stomatal conductance (g_s_) values noticed in our trial agree with those reported by Girona et al. (1994); their trial tested different irrigation regimes applied to “Pauetet” (Spanish cultivar). Comparable outcomes have also been reported by Pasqualotto et al. (2018); they noticed trees of hazelnut cultivars “Ennis” and “Tonda di Giffoni” located in France and Australia, respectively, were characterized by mean g_s_ values of about 200 mmol/m^2^/s. Considering that in our work the highest values of g_s_ were observed in leaves of “Negret N-9” grafted on “Dundee” (303.9 mmol/m^2^/s in late spring 2015), it suggests that this grafting combination promote the leaf stomata activity in comparison to other grafting combinations and self-rooted trees of the same cultivar. This hypothesis is also supported by the measurements of stem water potential carried out at the same time as those of g_*s*_. Although all plants were well-watered and stem water potential data are quite similar, plants grafted on “Dundee” showed the best values during the season.

From the present trial, we can point out that “Negret N-9” own-rooted YE was higher than “Negret N-9” grafted trees. Nevertheless, the own-rooted trees were small and weak, and they were thus more susceptible to abiotic and biotic stress. Considering the high YE of this cultivar, it could be thought that hazelnut production of grafted trees could be increased by forcing the tree with growing techniques. The implications for the future could be new layouts, different use of water and nutrients, etc. since few pieces of information about “Dundee” and agronomical interventions (fertilization and water supply) are known.

The leaf drop at the end of the growing season over the period of investigation were influenced by the grafting combination and the self-rooted trees showed the earliest leaf drop incidence in the October–December period, and this is in contrast to the trees grafted onto rootstock “Newberg” that were characterized by the slowest loss of leaves. The other grafting combinations showed an intermediate leaf drop rate. This is a relevant trait since the trees with slower leaf drop may maintain the photosynthetic apparatus and their biological functions longer during the growing season, which is reflected in a greater potential for capturing nutrients for a longer time and storing reserves in their root systems, as it has been noticed recently (Rovira, 2021).

Some other trials aiming to develop suitable rootstocks to graft the main hazelnut cultivars starting from pre-selection of *C. colurna* seedlings, *C. avellana* seedlings, and hybrids of *C. colurna* × *C. avellana* are still in progress in different hazelnut research centers in Chile, Italy, Romania, and Spain. The expected outputs of these trials, together with those obtained in the other few experiments available on the subject, will help to develop a primary hazelnut chain based on the growth of grafted and single-trunk trees replacing bush-trained trees, which is already underway in Spain for new plantings.

## Data Availability Statement

The raw data supporting the conclusions of this article will be made available by the authors, without undue reservation.

## Author Contributions

MR designed and carried out the experiments, field data, and laboratory data. JH was responsible for the agronomic management of the trail. JR designed and analyzed the physiological data. CS carried out some laboratory analysis and helped to draft the manuscript. VC revised the results and helped with the discussion. AR carried out the agronomic and laboratory statistical analysis and helped with the discussion. All authors contributed to the writing and review of the manuscript.

## Conflict of Interest

The authors declare that the research was conducted in the absence of any commercial or financial relationships that could be construed as a potential conflict of interest.

## Publisher’s Note

All claims expressed in this article are solely those of the authors and do not necessarily represent those of their affiliated organizations, or those of the publisher, the editors and the reviewers. Any product that may be evaluated in this article, or claim that may be made by its manufacturer, is not guaranteed or endorsed by the publisher.

## Supplementary Material

Supplementary Figure 1 Data of maximum and minimum air temperature and rainfall, collected by the thermometric station over the period 2003–2012.
