# Supplementary figures and images for: Agronomical and Physiological Behavior of Spanish Hazelnut Selection “Negret-N9” Grafted on Non-suckering Rootstocks
Source: Front Plant Sci. 2022 Feb 1;12:813902. doi: 10.3389/fpls.2021.813902 (PMC8845479; doi:10.3389/fpls.2021.813902)

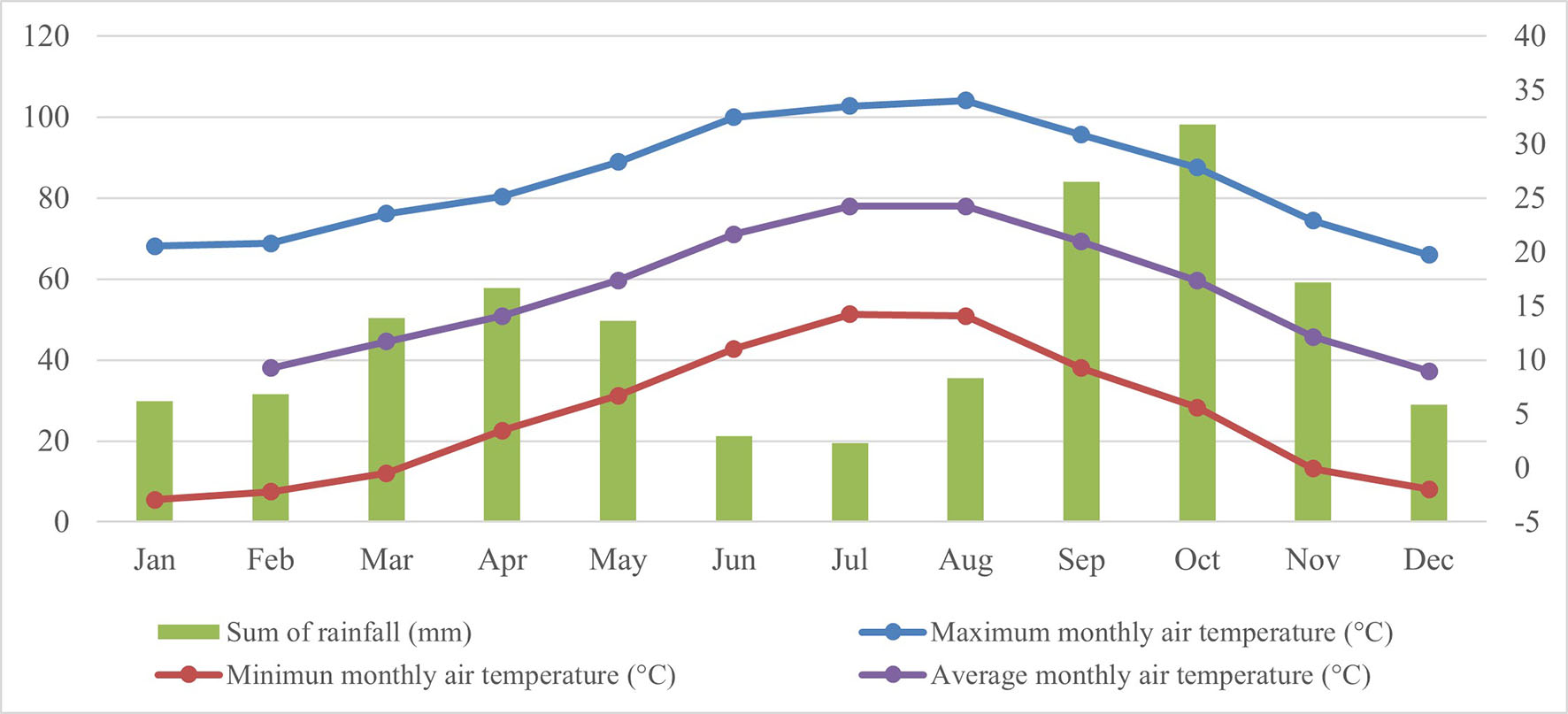

Supplement: Supplementary Figure 1 — Data of maximum and minimum air temperature and rainfall, collected by the thermometric station over the period 2003–2012. [file Image_1.JPEG]
